# Ranking relations between diseases, drugs and genes for a curation task

**DOI:** 10.1186/2041-1480-3-S3-S5

**Published:** 2012-10-05

**Authors:** Simon Clematide, Fabio Rinaldi

**Affiliations:** 1 Institute of Computational Linguistics, University of Zurich, Binzmühlestrasse 14, 8050 Zurich, Switzerland

## Abstract

**Background:**

One of the key pieces of information which biomedical text mining systems are expected to extract from the literature are interactions among different types of biomedical entities (proteins, genes, diseases, drugs, etc.). Several large resources of curated relations between biomedical entities are currently available, such as the Pharmacogenomics Knowledge Base (PharmGKB) or the Comparative Toxicogenomics Database (CTD).

Biomedical text mining systems, and in particular those which deal with the extraction of relationships among entities, could make better use of the wealth of already curated material.

**Results:**

We propose a simple and effective method based on logistic regression (also known as maximum entropy modeling) for an optimized ranking of relation candidates utilizing curated abstracts. Furthermore, we examine the effects and difficulties of using widely available metadata (i.e. MeSH terms and chemical substance index terms) for relation extraction. Cross-validation experiments result in an improvement of the ranking quality in terms of AUCiP/R by 39% (PharmGKB) and 116% (CTD) against a frequency-based baseline of 0.39 (PharmGKB) and 0.21 (CTD). For the TAP-10 metrics, we achieve an improvement of 53% (PharmGKB) and 134% (CTD) against the same baseline system (0.21 PharmGKB and 0.15 CTD).

**Conclusions:**

Our experiments with the PharmGKB and the CTD database show a strong positive effect for the ranking of relation candidates utilizing the vast amount of curated relations covered by currently available knowledge databases. The tasks of concept identification and candidate relation generation profit from the adaptation to previously curated material. This presents an effective and practical method suitable for conservative extension and re-validation of biomedical relations from texts that has been successfully used for curation experiments with the PharmGKB and CTD database.

## Background

The wealth of published information in the biomedical domain is at the same time an opportunity and a challenge. Accessing this information, and making sense of it, becomes an increasingly difficult task which requires a considerable expertise. In order to help the biologists quickly locate the essential information that they need, different organizations provide curated databases, which organize the available knowledge about a particular specific subject, for example UniProt/SwissProt [1] is one of the most authoritative resources concerning proteins, BioGrid [2] is the broadest database describing gene and protein interactions. Most reference databases are created and maintained using a very costly and expensive manual curation procedure, which involves highly skilled professionals. It has been observed already a few years ago that such an approach is not sufficiently efficient in order to cope with the increasing quantity of published results [3]. In order to support this process, researchers are turning their attention to text mining methodologies, not with the aim of replacing manual curation, which we consider not possible in the foreseeable future, but rather with the aim of providing tools that can make the curation process more efficient. Clearly such tools will need to be tailored to the specific task or database where they are going to be deployed, however some major tendencies are already clear and will shape the future development of the field. Some of the fundamental tasks that text mining systems are required to deal with are: term recognition, entity identification and the detection of important relations between entities.

The text mining community has been organizing a number of shared tasks aiming at providing an infrastructure for the comparative evaluation of different text mining technologies. One such task, which is of particular relevance to the work described in this paper, is the protein-protein interaction task which took place in the 2006 and 2009 editions of the BioCreative competitive evaluations [4,5]. The organizers provide a collection of annotated documents as a training dataset (typically derived from one of the curated databases) and a separate collection of unannotated documents as a test dataset. Participants have a limited time frame to process the training data and deliver results back to the organizers, who will then score these results against a previously withheld gold standard, using a set of metrics suited to the task. In this paper we focus on a different type of relations, namely those among genes, drugs/chemicals and diseases, and we use information derived from the PharmGKB database [6,7] and the CTD database [8] as our gold standard. These gold standards could be used in a text mining task analogous to the protein-protein interaction task defined in the BioCreative competitions.

We propose and evaluate a simple and practical method to achieve a high-quality ranked list of candidate relations based on the output of a term recognizer. Once entities have been identified, candidate relations can be generated with simple techniques, for example, co-occurrence within the same text span. However, such candidates would be too numerous to be useful, so proper ranking techniques are necessary in order to render these results accessible and really useful for a curation task. We use a machine learning approach suited for reranking of candidate relations by applying a maximum entropy method that integrates information from the vast amount of already curated relations from the PharmGKB and the CTD. This paper concludes with a brief overview of an integrated curation environment where the results described in the paper are applied.

## Methods

First we give a proper characterization of the resources and the gold standard data derived from the PharmGKB and the CTD databases. Next we present the evaluation measures and tools used for the experiments. Then we continue to describe our methods for term recognition, entity scoring, relation extraction and relation candidate ranking.

### Resources

In order to perform simple and replicable experiments we refrain from more sophisticated and resource-intensive entity recognition approaches and do not use any external database of names and identifiers, for instance, by leveraging synonyms from the UMLS [9] or BioPortal [10]. Instead we restrict the terminological dictionaries to the ones provided by the PharmGKB resp. the CTD that can be downloaded in a plain textual format. These resources include terms used in the curated papers and their unique identifiers for each corresponding entity. For the PharmGKB, we have 30351 terms (2986 IDs) for drugs, 28633 terms (3198 IDs) for diseases, 176366 terms (28633 IDs) for genes. For the CTD, we have 388384 terms (101030 IDs) for chemicals, 69483 terms (9657 IDs) for diseases, 711631 terms (79837 IDs) for genes. The terms for chemicals and disease of the CTD are largely from MeSH. The relationship data as available from the databases are represented as binary combination between two typed identifiers, supplemented with additional information regarding the type of evidence supporting the relationship. For all experiments described in this paper, we limit the set of relations to the ones based upon manually curated evidence from PubMed. In particular, we do not use inferred relations from the CTD and automatically created relation annotations from the PharmGKB, which were accessible in the past through their web interface.

From the PharmGKB, we get 26122 binary relations, which are based upon 5062 distinct PubMed articles. However, the number of relations attributed to an article varies strongly between just 1 up to 600 relations per article. Given that we consider only abstracts and not full-text, the task of extracting more than two dozens of relations seems not realistic. We therefore decided to restrict the data set for our experiments to all articles containing at most 20 relations. The resulting 4658 articles, which we then used for our experiments, contain 14825 relations. The source databases include some reflexive relations, i.e. relations between identical concepts, which we removed from our dataset. Table 1 shows the exact distribution of all relation types in our experimental data set split up by the number of relations in an article. As can be seen there, relations between the three different entity types, i.e. diseases (henceforth "Di"), drugs ("Dr") and genes ("Ge"), do not occur uniformly. In our data set, about 42% of all relations are of type Drug-Gene (Dr-Ge), about 37% of type Disease-Gene (Di-Ge) and only 18% of type Disease-Drug (Di-Dr). Relations between entities of the same type do exist, but they are marginal and contribute only about 3% of all relations.

**Table 1:** Distribution of relations per article in experimental data sets.

PharmGKB data set													
**#Rels**	**Di-Di**		**Di-Dr**		**Di-Ge**		**Dr-Dr**		**Dr-Ge**		**Ge-Ge**		**all**
**per Art**	**abs**	**rel**	**abs**	**rel**	**abs**	**rel**	**abs**	**rel**	**abs**	**rel**	**abs**	**rel**	**sum**

1	2	0.1	129	6.6	842	43.0	29	1.5	938	47.9	19	1.0	1959
2	6	0.5	138	10.9	484	38.1	18	1.4	611	48.1	13	1.0	1270
3	1	0.0	705	26.5	925	34.8	12	0.5	993	37.4	22	0.8	2658
4	9	1.2	98	13.1	231	30.9	21	2.8	372	49.7	17	2.3	748
5	0	0.0	397	24.3	575	35.2	15	0.9	636	38.9	12	0.7	1635
6	7	1.1	62	9.6	237	36.6	19	2.9	301	46.5	22	3.4	648
7	1	0.1	154	20.0	293	38.1	6	0.8	296	38.4	20	2.6	770
8	0	0.0	155	18.5	334	39.8	17	2.0	320	38.1	14	1.7	840
9	12	1.6	153	19.8	283	36.6	12	1.6	279	36.0	35	4.5	774
10	10	4.2	32	13.3	74	30.8	0	0.0	114	47.5	10	4.2	240
11	1	0.1	205	28.2	236	32.5	4	0.6	270	37.2	10	1.4	726
12	12	3.4	67	19.3	87	25.0	11	3.2	165	47.4	6	1.7	348
13	0	0.0	47	18.1	100	38.5	6	2.3	107	41.2	0	0.0	260
14	0	0.0	52	19.5	118	44.4	0	0.0	93	35.0	3	1.1	266
15	7	1.7	77	18.3	144	34.3	0	0.0	189	45.0	3	0.7	420
16	0	0.0	40	19.2	100	48.1	0	0.0	68	32.7	0	0.0	208
17	0	0.0	39	17.6	51	23.1	7	3.2	106	48.0	18	8.1	221
18	0	0.0	2	1.2	56	34.6	0	0.0	100	61.7	4	2.5	162
19	0	0.0	36	23.7	59	38.8	0	0.0	57	37.5	0	0.0	152
20	0	0.0	127	24.4	203	39.0	4	0.8	166	31.9	20	3.8	520

TOTAL	68	0.5	2715	18.3	5432	36.6	181	1.2	6181	41.7	248	1.7	14825

**CTD data set**													

**#Rels**			**Di-Dr**		**Di-Ge**				**Dr-Ge**				**all**
**per Art**			**abs**	**rel**	**abs**	**rel**			**abs**	**rel**			**sum**

1			1482	23.3	1333	21.0			3539	55.7			6354
2			2454	21.9	1539	13.7			7219	64.4			11212
3			1806	17.6	1154	11.3			7294	71.1			10254
4			1717	15.5	994	9.0			8357	75.5			11068
5			1144	15.3	507	6.8			5824	77.9			7475
6			1248	14.5	525	6.1			6855	79.5			8628
7			578	12.7	270	6.0			3688	81.3			4536
8			648	14.1	225	4.9			3719	81.0			4592
9			396	14.4	165	6.0			2184	79.6			2745
10			285	12.7	88	3.9			1867	83.3			2240
11			193	13.7	93	6.6			1122	79.7			1408
12			203	15.1	63	4.7			1078	80.2			1344

TOTAL			12154	16.9	6956	9.7			52746	73.4			71856

This table shows how many relations between which entities occur per article in both data sets. PharmGKB has some relations between entities of the same type. CTD contains only relations between entities of different types. In order to keep the tables of both databases easily comparable, entities of type "chemical" from CTD are labeled with "Dr" (drugs).

From the CTD, we get 294151 binary relations, which are based upon 27960 distinct PubMed articles. However, the number of relations attributed to an article varies strongly between just 1 up to more than 9500 relations per article. Given the fact that the CTD provides a lot more gold standard relations we restrict the data set for our experiments with the CTD to all articles containing at most 12 relations. The remaining 23257 articles contain 71856 relations. The lower part of Table 1 shows the exact distribution for these relations for the CTD. In this data set, about 73% are of type Chemical-Gene (for the sake of comparability with the PharmGKB, we recast the CTD entity type "chemical" as "Dr"), about 17% are of type Disease-Chemical (Di-Dr), and only 10% are of type Disease-Gene (Di-Ge). The CTD contains no relations between entities of the same type.

### Measures and tools for evaluation

The format of the relationship file provided by the knowledge bases lends itself to an easy transformation into a format equivalent to the one used for the protein-protein interaction (PPI) task of BioCreative II.5 [5]. Given a text mining tool which can produce a ranked list of gene/drug/disease relations, it becomes then possible to score these results against the knowledge base data by using a scoring tool provided by the BioCreative organizers.

The BioCreative PPI evaluation tool returns results according to the standard metrics used in information retrieval (Precision, Recall, F-Measure) as well as a more novel measure called "AUC iP/R" (area under the curve of the interpolated precision/recall graph). The AUC iP/R measure (not to be confused with the more frequently used "AUC of the ROC curve" metric) provides an indication of the quality of the ranking of the candidate relations. The intuitive idea is that, given equivalent P/R/F figures, correct predictions which occur towards the top of the ranked list are more useful than the ones which are lower in the ranking. The implicit assumption is that a curator could use the ranking to decide where to stop looking at the candidate results, therefore a better ranking provides a better user experience. The AUC iP/R curve is defined in [11], a detailed operative description of AUC iP/R, as used in the BioCreative evaluations, can be found at http://www.biocreative.org/tasks/biocreative-ii5/biocreative-ii5-evaluation/.

A recently proposed alternative evaluation measure for ranked results is the "Threshold Average Precision" (TAP-k) [12], which (in slightly simplified terms) averages precision for the results above a given error threshold. The TAP-k metric is easier to interpret and also directly relevant for the end user, who in most cases would not be willing to inspect a long list of candidate relations containing many false positives. The TAP-k mirrors the fact that a curator will stop validating a list of ranked relation candidates after having rejected a certain number *k *of false positives. In our main experiments, we set *k *= 10.

Note, that the values of the evaluation metrics reported here are always macro-averaged, i.e. the mean of the evaluation score is computed separately for each article.

### Text processing and term recognition

For the experiments we use PubMed abstracts corresponding to the PubMed IDs mentioned by the relationship files from the knowledge bases. It would of course be desirable to work on full papers rather than abstracts, however, not all these publications are freely accessible, and most importantly, they are not available in a common format. The lack of a common format hinders the usability of full-text publications for practical text mining purposes, as it makes it more difficult to identify significant parts of the papers (e.g. results sections) or distinguish elements that require special processing (e.g. tables).

In the experiments, we apply the first processing steps of our OntoGene relation mining system (OG-RM) in order to annotate the input documents with the terminology provided by the respective knowledge bases. First, in the preprocessing stage, the PubMed XML is transformed into a custom XML format where sentences and tokens boundaries are identified using the LingPipe framework (for more information see http://alias-i.com/lingpipe). Second, the OntoGene pipeline proceeds with a step of term annotation [13,14]. In order to account for possible surface variants and in order to allow for partial matches, a normalization step is included in the annotation procedure. The annotations generated by the OntoGene pipeline can then be used to generate candidate relations using a number of different criteria. Since each token in the OntoGene annotation framework is assigned a unique identifier, extracted terms can be related back to their position in the text.

#### Selecting textual material and metadata

The experimental settings described below vary according to the amount of metadata that is included in the text mining process:

1. only title and abstract of the article are used (henceforth t);

2. additionally to t the names in the chemical substance list of an abstract are used (henceforth tc);

3. additionally to t the MeSH descriptors and their qualifiers are used (henceforth tm);

4. all possible information is used (henceforth tmc).

The motivation for the inclusion of metadata such as MeSH or chemical substance lists is an improved recall of the term recognition. Table 2 shows the exact improvement for our experimental data set from the PharmGKB. Diseases have the lowest coverage of 67% and profit, as expected, substantially from the inclusion of MeSH terms (+8%). Drug recognition improves using the list of chemical substances (+3%), but does not further improve by adding the MeSH terms. As our term recognizer is tuned towards the detection of proteins and genes, we reach the highest coverage for genes, as expected. Using the metadata still gives an improvement of 2%. For all entities we cover 74% with text only (t) and 78% using all metadata (tmc).

**Table 2:** Coverage of term recognition for concepts and relations in experimental data

PharmGKB data set									
		**t**		**tm**		**tc**		**tmc**	
**Entity**	**N**	**abs**	**rel**	**abs**	**rel**	**abs**	**rel**	**abs**	**rel**

Di	3830	2550	66.58	2872	74.99	2557	66.76	2872	74.99
Dr	4751	3527	74.24	3632	76.45	3668	77.20	3668	77.20
Ge	7522	5838	77.61	5930	78.84	5989	79.62	5994	79.69

TOTAL	16103	11915	73.99	12434	77.22	12214	75.85	12534	77.84

Di-Di	68	22	32.35	24	35.29	22	32.35	24	35.29
Di-Dr	2715	1279	47.11	1484	54.66	1326	48.84	1494	55.03
Di-Ge	5432	3102	57.11	3555	65.45	3181	58.56	3585	66.00
Dr-Dr	181	128	70.72	132	72.93	135	74.59	135	74.59
Dr-Ge	6181	3858	62.42	4016	64.97	4097	66.28	4099	66.32
Ge-Ge	248	141	56.85	142	57.26	145	58.47	145	58.47

TOTAL	14825	8530	57.54	9353	63.09	8906	60.07	9482	63.96

**CTD data set**									

		**t**		**tm**		**tc**		**tmc**	
**Entity**	**N**	**abs**	**rel**	**abs**	**rel**	**abs**	**rel**	**abs**	**rel**

Di	12639	6939	54.90	9502	75.18	6941	54.92	9502	75.18
Dr	38523	27541	71.49	29531	76.66	30119	78.18	30129	78.21
Ge	39150	28389	72.51	28975	74.01	29169	74.51	29199	74.58

TOTAL	90312	62869	69.61	68008	75.30	66229	73.33	68830	76.21

Di-Ge	6956	4117	59.19	5100	73.32	4163	59.85	5126	73.69
Dr-Di	12154	5335	43.90	8219	67.62	5700	46.90	8356	68.75
Dr-Ge	52746	31015	58.80	33971	64.40	34832	66.04	34883	66.13

TOTAL	71856	40467	56.32	47290	65.81	44695	62.20	48365	67.31

Distribution of identifiable gold standard concepts and relations given the output from our term recognizer and split according to the inclusion of metadata: text only (t), text and MeSH terms (tm), text and chemical substance list (tc), text and all metadata (tmc).

The lower half of Table 2 shows the corresponding numbers for the CTD data. Diseases again have the lowest coverage for text only (55%) and profit heavily from MeSH terms (+20%). Chemical coverage improves mostly by information from the chemical substance list (+7%), but the MeSH terms add also most of the important information. Detection of genes based on the text only is again high and improves slightly (+2%) when metadata is added, but remains clearly beyond the recognition rate achieved for the PharmGKB. Regarding the coverage of relations, we see an improvement of 6% in the case of the PharmGKB, and almost 11% for the CTD.

### Relation extraction and relation ranking

There are several ways in which the entities recognized in an abstract can be combined, for example by co-occurrence in the same sentence, or by using a set of syntactic filters as done in our previous work on protein-protein interactions [15,16]. The approach which delivers the maximal recall is to generate all pairwise undirected combinations of all entities identified in the abstract.

As shown in Table 2 for the PharmGKB, this approach can deliver a recall of 58% using only text (t), 63% using additionally MeSH (tm) and 64% using all the metadata (tmc). Note, that the upper limit of the recognition rate varies strongly by the type of entities involved in a relation, disease-drug relations have an unexpected low upper limit in PharmGKB. The lower part of Table 2 shows similar numbers for the CTD, i.e., a recall of 56% (t), 66% using MeSH (tm) and 67% using chemical substance lists as well (tmc). Considering that only abstracts were used, this seems a reasonable term recognition coverage for our experiments. However, this approach will massively overgenerate, therefore ranking of the results becomes absolutely necessary.

In order to reduce the overgeneration of relation candidates, one could limit the set of candidate relations to entities that co-occur at least once in the same sentence. However, experiments we performed with such co-occurrence limits resulted in inferior performance. Table 3 explains this rather unexpected effect to some degree: for about 30% of the relations from the gold standard where our term recognizer is able to detect both entities in the article there is no sentence containing a hit for both entities in the PharmGKB. For the CTD, about 32% of the gold standard relation cannot be found in the same sentence. A term recognizer with improved acronym detection and coreference resolution may alleviate this problem.

**Table 3:** Occurrence of gold standard relations in the same sentence

PharmGKB data set					
	**In Same Sentence**		**In Diff. Sentences**		
**Relation**	**Absolute**	**Relative**		**Relative**	**Total**

Dr-Dr	111	86.7	17	13.3	128
Ge-Ge	113	80.1	28	19.9	141
Dr-Ge	2 895	75.0	963	25.0	3 858
Di-Ge	2 009	64.8	1 093	35.2	3 102
Di-Dr	816	63.8	463	36.2	1 279
Di-Di	13	59.1	9	40.9	22

All	5 957	69.8	2 573	30.2	8 530

**CTD data set**					

	**In Same Sentence**		**In Diff. Sentences**		
**Relation**	**Absolute**	**Relative**		**Relative**	**Total**

Di-Ge	3 457	75.6	1 118	24.4	4 575
Dr-Ge	23 123	67.0	11 365	33.0	34 488
Dr-Di	3 948	66.6	1 982	33.4	5 930

All	30 528	67.9	14 465	32.1	44 993

Distribution of all gold standard relations where both entities could be identified by our term recognizer. An occurrence of a relation is categorized as "In Same Sentence" if there exists at least one sentence in a given abstract where both entities co-occur. An occurrence of a relation is categorized as "In Different Sentences" if both entities can be found in a given abstract but never co-occur together in the same sentence. For these tables metadata such as MeSH terms and chemical substance lists were not included.

#### Ranking relations by frequency

A baseline ranking of all candidate relations of an abstract can be generated on the basis of the number of occurrences of the respective entities:

$$relscore\left(e_{1},\;e_{2}\right)=\;\frac{f\left(e_{1}\right)+f\left(e_{2}\right)}{f\left(E\right)}$$

where *f*(*e*_1_) and *f*(*e*_2_) are the number of times the entities *e*_1 _and *e*_2 _are observed in the abstract, while *f*(*E*) is the total count of all entities in the abstract. Once a score is assigned to each candidate pair, it is possible to filter out the most unlikely candidates, either by setting a threshold value for the score, or by selecting only the N-best candidates. Using one of these filtering techniques will result into variable values of Precision, Recall and F-Measure, depending on the exact value of the score threshold, or N parameter.

#### Title occurrence boosting

We know from our previous experiments [15] that giving a "boost" to the entities contained in the title can produce a measurable improvement of ranking of the results (measured by the AUC or TAP metrics). We have empirically verified that a sensible boost for abstracts is around 10. This is equivalent to counting the entities in the title ten times. In the rest of this paper, boosted frequencies of entities are expressed as *f_b_*(*e*).

The baseline approach for relation ranking described above will be referred to as m0 in the rest of this paper.

#### Preferring relations between unequal types

As shown in Table 1, relations between entities of the same type occur far less often in the PharmGKB than relations between different types. We can model this empirical fact by applying a type preference coefficient to the relation score that affects relations between entities of the same type. An empirically set coefficient of 1/10 proved to be useful:

$$typepref\left(e_{1},e_{2}\right)=\left\{\begin{array}{cc} 0.1\; & \text{if both entities have the same type}\\ 1\; & \text{otherwise}. \end{array}\right)$$

In the experiments described in Section 'Results and discussion', we express the application of the type coefficient in the following way:

1. no type preference applied (henceforth e0);

2. type preference applied (henceforth e1).

Additionally, we experimented with using the relative frequency of a relation type taken from the training set as a type preference coefficient. Because results deteriorated consistently when using this setting, we do not take it into account for the evaluation. Note that the CTD does not contain relations between equal types, therefore the experimental settings for the CTD do not vary this parameter.

#### Scoring entities for being part of curated gold standard relations

The ranking of relation candidates using a simple frequency-based confidence score derived from textual evidence can be further optimized if we apply a supervised machine learning method (in our case a Maximum Entropy technique) that models the relevance of an entity using the curated relations from the gold standard and the documents where these relations occur. In our experiments described below, results computed using this technique will be tagged as m1.

There are two motivations for scoring concepts with regard to relation ranking: First, we want to identify automatically the false positive entities that our term recognizer detects in order to penalize them. The term recognizer eagerly modifies term entries from the dictionary while matching, i.e. material is removed from an entry in the term dictionary in order to allow for partial matches, or on-the-fly acronyms are created. For instance, the term form "neuronal" may be identified as the genes PA134898200, PA134924203, PA134896732 from the term database because they have "neuronal protein" as one of their lexical entries. Once identified such false positive partial matches could be ruled out by ad-hoc rules. However, for different terminological resources different rules may be necessary. We regard a general approach that works independently from the used terminological resources and that achieves an automatic adaptation as highly beneficial. In order to deal with such cases, we need not only to condition on the entities, but also on their textual representation.

Second, we need to adapt to highly ranked false positive relations which are generated by our frequency based approach by frequent but irrelevant entities. The goal is to identify some global (dis)preference that can be found in the PharmGKB or the CTD relationships.

#### Normalizing term forms

For a precise description of the ME-optimized ranking approach, we need to introduce some notation. In the following, the notation *t *refers to a normalized textual form of a recognized term. In the experiments, we vary four levels of normalization:

1. no normalization except lower-case initial characters (henceforth n0);

2. lower-case characters and some punctuation removed: '\\()\ /- (henceforth n1);

3. lower-case characters and only alphanumeric characters retained in tokens (henceforth n2);

4. same as 3, but token boundaries are removed (henceforth n3).

For instance, "Fc ( gamma ) - receptor" is normalized to "fc gamma receptor" in mode n1, in mode n3 we get "fcgammareceptor". Multiple spaces resulting from the deletion of characters are squeezed into one. [17] have shown that the removal of punctuation symbols does not harm the term recognition quality. The combination of a term *t *and one of its valid entities *e *is noted as *t*:*e*.

#### Applying counting caps

Because term frequency in an article seems crucial for an estimation of the relevance of a concept, we condition valid term-entity combinations additionally on their number of occurrences in an article. In order to reduce the resulting problem of data sparseness we apply different upper limits (so-called caps) on the raw frequencies:

$$f^{c}\left(t:e\right)=\left\{\begin{array}{cc} cap\; & \text{if }f\left(t:e\right)\geq cap\\ f\left(t:e\right)\; & \text{otherwise}. \end{array}\right)$$

In the experiments, we test different settings:

1. *cap *= ∞, i.e no cap is used (henceforth c0);

2. *cap *= 1, i.e. a term-entity is present or not (henceforth c1);

3. *cap *= 3, *cap *= 6, *cap *= 9 (henceforth c3, c6, c9).

### Estimating gold probabilities

Next we define a predicate *gold*(*A*, *e*) which is true (i.e. 1) for an article *A *if there is at least one relation in the gold standard where entity *e *is part of, and false (i.e. 0) otherwise. Using the notions defined beforehand, we specify the overall probability of an entity *e *of being part of a gold relation given the entity *e*, a term form *t*, and their frequency *f ^c ^*(*t*:*e*) in article *A*:

$$P\left(gold\left(A,e\right)=1|e,t,f^{c}\left(t:e\right)\right)$$

We estimate *P*(*gold*(*A*, *e*) = 1 | *e*, *t*, *f ^c^*(*t *: *e*)) with the help of the Maximum Entropy Modeling tool *megam *[18] using the recognized terms of the abstracts from a training set together with the gold standard information from the same document set. Technically, each value *e*, *t*, *f^c^*(*t*:*e*) from an article serves as a joint feature for the maximum entropy classifier and the value of *gold*(*A*, *e*) as its binomial class, i.e. a number between 0 and 1. This numeric value will be predicted by the model when features from unseen articles are presented. The model of a maximum entropy classifier consists of a weight for each feature of the training material. Formally, a conditional Maximum Entropy Model (aka. Logistic Regression) has the following exponential form:

$$\begin{array}{llll} p_{\lambda }\left(y|x\right) & =\frac{1}{Z\left(x\right)}\text{exp}\left(\underset{i}{\sum }\lambda_{i}F_{i}\left(x,y\right)\right)\; & & \;\\ Z\left(x\right) & =\underset{y}{\sum }\text{exp}\left(\underset{i}{\sum }\lambda_{i}F_{i}\left(x,y\right)\right),\; & & \;\\ & \; & \end{array}$$

where *y *is the joint feature *e*, *t*, *f ^c^*(*t *:*e*) and *x *is the value of the gold predicate *gold*(*A*, *e*). In the formula, we designate Maximum Entropy features by *F_i _*as the notation *f *is used for frequencies in this paper. The Maximum Entropy Modeling tool iteratively optimizes the feature weights λ in such a way that they maximize the conditional log-likelihood of the training material. There are two practical reasons for our choice for Maximum Entropy modeling: Firstly, this classifier does not suffer when dependent features are used, such as our smoothing features introduced below. Therefore, an approach as for instance a Naive Bayes classifier is not generally feasible for our method. Secondly, the Maximum Entropy tools performs very efficiently with ten thousands of features and it requires no parameter tuning as for example most Support Vector Machine tools.

#### Smoothing counts

For features not present in the training material there are no weights available. In order to reduce the resulting sparse data problem, we apply a smoothing method that works as follows: for each feature *e,t*, *f ^c^*(*t*:*e*) add all additional features e, *t*, *n *with *f ^c^*(*t*:*e*) >*n *≥ 1. In our experiments described in the section 'Results and discussion', we evaluate the effect of feature smoothing as follows:

1. do not smooth (henceforth s0);

2. apply smoothing (henceforth s1).

In the case of applying a cap of 1 (i.e. c1), smoothing (i.e. s1) is not necessary and the equation for the gold probability simplifies to the following:

P(gold(A,e)=1|t:e)

For unseen terms *t*, i.e. terms not present in the training data, the maximum entropy classifier assigns a default probability based on the distribution of all training instances. However, we can specify better back-off probabilities if we take into account the admissible entity/entities *e *of term *t*. Our current back-off model works as follows: if the entity *e *of an unseen *t *is seen in the article, the averaged probability of all seen term-entity pairs is used. Otherwise, the averaged probability of all entities of the same type as *e *is used.

#### Scoring entities

Finally, the resulting score of an entity *e *in an article *A *is the sum of the boosted term frequency weighted by the gold probability:

$$score\left(e\right)=\underset{t:e\;\in A}{\sum }f_{b}\left(t:e\right)\times P\left(gold\left(A,e\right)=1|e,t,f^{c}\left(t:e\right)\right)$$

#### Scoring relations

Having determined the score of each entity *e*, we add them to a relation score similar to the baseline method:

$$relscore\left(e_{1},e_{2}\right)=\left(score\left(e_{1}\right)+score\left(e_{2}\right)\right)\times typepref\left(e_{1},e_{2}\right)$$

This simple relation score function has the disadvantage that a single entity score with a high value produces a high relation score even if the other entity has a very low entity score. As an alternative we use the harmonic mean of both entity scores in order to decrease the relation score of entity combinations with highly disparate entity scores.

$$relscore_{h}\left(e_{1},e_{2}\right)=2\times \frac{score\left(e_{1}\right)\times score\left(e_{2}\right)}{score\left(e_{1}\right)+score\left(e_{2}\right)}\times typepref\left(e_{1},e_{2}\right)$$

In the evaluation we encode the different relation score metrics as follows:

1. simple sum of entity scores (henceforth r0);

2. harmonic mean of entity scores (henceforth r1).

### Experimental settings at a glance

For the cross-validation experiments described in the next section, we vary the following settings:

• title and abstract (t), including MeSH (tm), including chemical substances (tc), including all metadata (tmc);

• no type preference coefficient (e0); preference coefficient for unequal type (e1);

• relation score as sum (r0) or as harmonic mean (r1) of entity score;

• baseline approach (no weighting of entities) (m0) vs. maximum entropy (ME) weighting (m1);

• normalization of term forms for ME: first letter in lower-case (n0), all characters in lower-case and some punctuation marks removed (n1), lower-case alphanumeric characters with spaces (n2), lower-case alphanumeric characters without spaces (n3);

• caps for ME features: no cap, i.e. raw counts (c0), cap of 1, 3, 6 or 9 (c*n*);

• smoothing of ME features is off (s0) or on (s1).

Note that the settings n, c and s are only meaningful for the ME approach. The baseline system as mentioned in the following section is identified by the settings t-e0-r0-m0-n0-c0-s0 or t-e0-r0-m0 for short. The setting e1 is only applicable to the PharmGKB.

## Results and discussion

In this section, we report on the systematic stratified 10-fold cross-validation evaluation using all different experimental settings mentioned in the preceding section. All numbers presented in this section are means of 10 different runs. Our data sets from the PharmGKB and the CTD were split into subsets stratified according to the number of relations per article. See Table 1 for the distribution of the frequency of relations per article. Note that we did not enforce a stratified distribution of different relation types in all subsets.

Taking into consideration all valid configurations of experimental settings leads to several hundred combinations to test for and to the same number of results to compare. For reasons of space we focus our presentation and discussion on the most important question to be answered by our results: which feature setting contributes how much performance increase to the baseline system or improvements thereof? We give a tabular overview of performance increase in terms of TAP-10 (Table 4) and AUCiP/R (Table 5) separately for the PharmGKB and the CTD.

**Table 4:** Evaluation of performance increase of TAP-10

PharmGKB data set								
**setting**	**mean**	**sd**	**Δ**	**Δabs**	**Δrel**	**p value**	**ΔCI*_l_***	**Δrel*_bs_***

t-e0-r0-m0-n0-c0-s0	0.27	0.010						
t-e1-r0-m0-n0-c0-s0	0.32	0.010	e1	+0.042	+15.3	9.8_-4_	+0.039	+15.3
t-e1-r0-m1-n0-c0-s0	0.35	0.010	m1	+0.030	+9.6	9.8_-4_	+0.028	+26.4
t-e1-r0-m1-n0-c1-s0	0.37	0.012	c1	+0.019	+5.5	9.8_-4_	+0.017	+33.4
t-e1-r1-m1-n0-c1-s0	0.39	0.012	r1	+0.028	+7.6	9.8_-4_	+0.025	+43.6
tmc-e1-r1-m1-n0-c1-s0	0.41	0.012	tmc	+0.016	+4.2	9.8_-4_	+0.011	+49.6
tmc-e1-r1-m1-n2-c1-s0	0.41	0.011	n2	+0.003	+0.7	9.8_-4_	+0.001	+50.6
tmc-e1-r1-m1-n2-c6-s1	0.42	0.009	c6-s1	+0.005	+1.1	9.8_-4_	+0.002	+52.3
tmc-e1-r1-m1-n3-c6-s1	0.42	0.009	n3	+0.002	+0.5	2.9_-3_	+0.001	+53.0

**CTD data set**								

**setting**	**mean**	**sd**	**Δ**	**Δabs**	**Δrel**	**p value**	**ΔCI*_l_***	**Δrel*_bs_***

t-r0-m0-n0-c0-s0	0.15	0.006						
t-r0-m1-n0-c0-s0	0.22	0.004	m1	+0.074	+49.5	9.8_-4_	+0.072	+49.5
t-r0-m1-n0-c1-s0	0.28	0.007	c1	+0.055	+24.7	9.8_-4_	+0.052	+86.5
tmc-r0-m1-n0-c1-s0	0.31	0.021	tmc	+0.029	+10.2	2.0_-3_	+0.030	+105.6
tmc-r1-m1-n0-c1-s0	0.32	0.021	r1	+0.014	+4.6	9.8_-4_	+0.013	+115.0
tmc-r1-m1-n3-c1-s0	0.34	0.005	n3	+0.019	+5.8	2.0_-__3_	+0.010	+127.5
tmc-r1-m1-n3-c3-s1	0.35	0.005	c3-s1	+0.010	+3.0	9.8 _-4_	+0:007	+134.4

These tables show the amount of performance increase by exchanging one experimental parameter by another parameter (or a parameter combination, if no single parameter exchange increases the performance). From one row of the table to the following row we select the parameter(s) that gives the highest performance increase as measured by the mean TAP-10 from the cross validation runs. The absolute performance increase (Δabs) and the relative increase (Δrel) between two adjacent rows are reported in the corresponding columns. A Wilcoxon signed rank test for dependent pairs is used to assess whether the improvement is significant or due to chance. The last column shows the relative increase compared to the baseline setting (Δrel*_bs_*).

**Table 5:** Evaluation of performance increase of AUCiP/R

PharmGKB data set								
**setting**	**mean**	**sd**	**Δ**	**Δabs**	**Δrel**	**p value**	**ΔCI*_l_***	**Δrel*_bs_***

t-e0-r0-m0-n0-c0-s0	0.39	0.015						
t-e1-r0-m0-n0-c0-s0	0.44	0.012	e1	+0.048	+12.1	9.8_-4_	+0.044	+12.1
t-e1-r0-m1-n0-c0-s0	0.49	0.014	m1	+0.044	+10.0	9.8_-4_	+0.042	+23.2
t-e1-r0-m1-n0-c1-s0	0.51	0.017	c1	+0.027	+5.5	9.8_-4_	+0.023	+30.0
tmc-e1-r0-m1-n0-c1-s0	0.53	0.015	tmc	+0.018	+3.4	9.8_-4_	+0.013	+34.5
tmc-e1-r1-m1-n0-c1-s0	0.54	0.015	r1	+0.012	+2.3	9.8_-4_	+0.010	+37.6
tmc-e1-r1-m1-n2-c1-s0	0.55	0.015	n2	+0.003	+0.6	9.8_-4_	+0.002	+38.4
tmc-e1-r1-m1-n2-c3-s1	0.55	0.013	c3-s1	+0.003	+0.5	1.5_-2_	+0.001	+39.1
tmc-e1-r1-m1-n3-c0-s1	0.55	0.014	n3-c0	+0.002	+0.4	1.4_-2_	+0.000	+39.7

**CTD data set**								

**setting**	**mean**	**sd**	**Δ**	**Δabs**	**Δrel**	**p value**	**ΔCI*_l_***	**Δrel*_bs_***

t-r0-m0-n0-c0-s0	0.21	0.008						
t-r0-m1-n0-c0-s0	0.30	0.006	m1	+0.090	+42.4	9.8_-4_	+0.087	+42.4
t-r0-m1-n0-c1-s0	0.37	0.009	c1	+0.072	+23.7	9.8_-4_	+0.068	+76.1
tmc-r0-m1-n0-c1-s0	0.41	0.026	tmc	+0.040	+10.6	2.0_-3_	+0.042	+94.9
tmc-r0-m1-n3-c1-s0	0.43	0.006	n3	+0.018	+4.3	2.9_-3_	+0.005	+103.3
tmc-r1-m1-n3-c1-s0	0.45	0.007	r1	+0.015	+3.5	9.8_-4_	+0.013	+110.4
tmc-r1-m1-n3-c3-s1	0.46	0.007	c3-s1	+0.012	+2.8	9.8_-4_	+0.009	+116.2

This table gives the corresponding overview of feature-wise performance increase as in Table 4. See Table 4 for a detailed explanation on the interpretation of the columns.

These tables give a concise compilation of the following information:

• The mean and standard deviation (noted as "sd") from the 10-fold cross-validation results of a given setting.

• The single experimental parameter setting that needs to be changed in order to achieve the highest performance increase. Only if no single parameter with better performance can be found, two parameters (or more) may be changed at once.

• The absolute (Δabs) and relative (Δrel) amount of performance improvement.

• The statistical significance of the improvement given as the p value of a Wilcoxon signed rank test for dependent pairs.

• An estimate of the minimal improvement expected in 95% of all cases, i.e. the lower limit of the 95% confidence interval (ΔCI*_l_*) taken from the Wilcoxon test.

• Finally, the relative performance improvement in comparison to the baseline (Δrel*_bs_*).

The Wilcoxon signed rank test for dependent pairs is used to assess whether the improvement is significant or due to chance. The experimental setting of 10-fold cross validation leads to a small sample size and additionally the differences of means used for this kind of comparison are not always normally distributed in our data. In order to be able to apply the same significance test to all settings, such a non-parametric significance test is more appropriate than the parametric t-test. The p values and the non-parametric 95% confidence interval are exact values and not normal approximations (the test for improvement is one-sided and therefore only the lower limit of improvement is actually shown in the tables). We use the function wilcox.exact() from the library exactRankTests of the statistical software framework R. See the documentation for more technical details. Further discussion of the appropriateness of significance tests on results gained by cross-validation can be found in [19,20].

**Figure 1:**
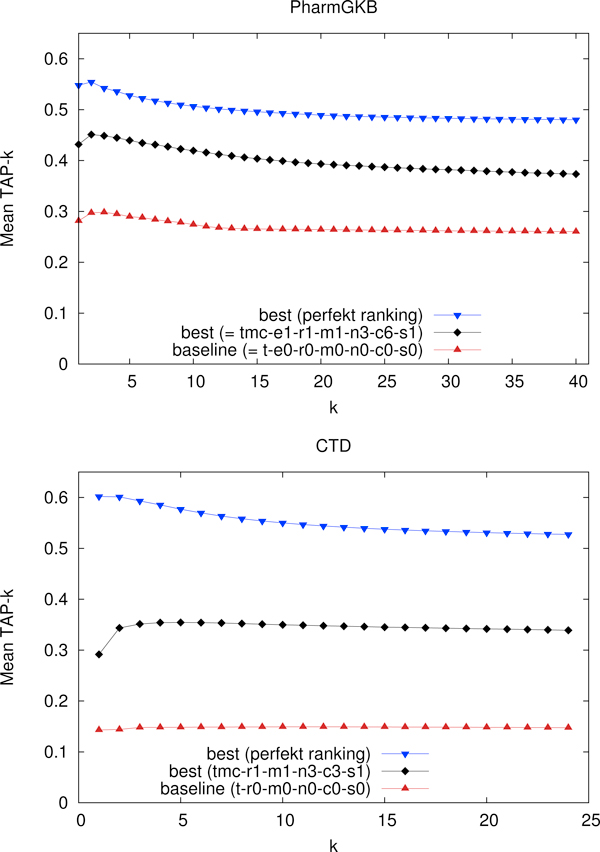
**Evaluation of TAP-k**. Mean macro-averaged results from the TAP-k tool. The horizontal axis shows the k value limiting the number of results that are evaluated by the tool by specifying a threshold on the number of false positives.

**Figure 2:**
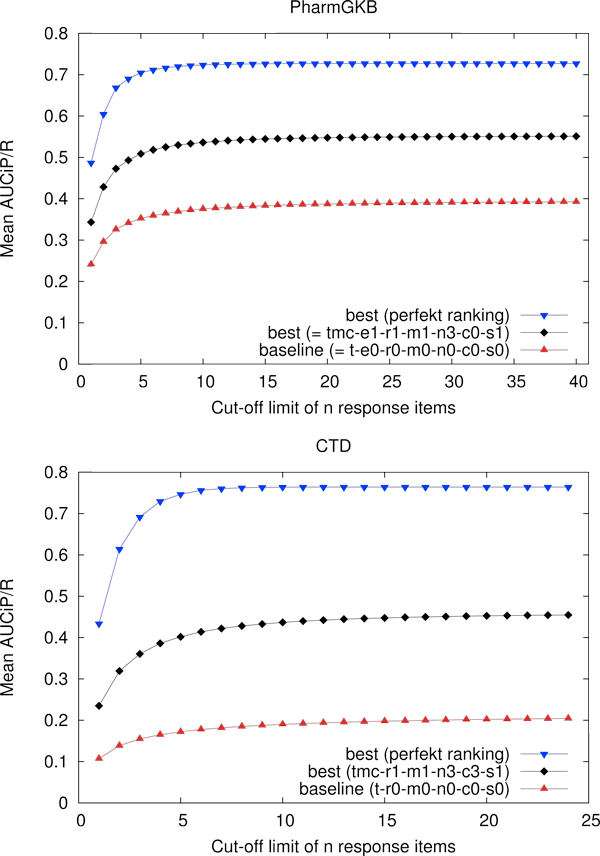
**Evaluation of AUCiP/R**. Mean macro-averaged results for different cut-off limits using the BioCreative evaluation tool. The horizontal axis shows the cut-off value limiting the number of hits that are evaluated by the tool. The vertical axis shows macro averaged results of AUC iP/R for our different settings.

Although the tables mentioned above iteratively answer the question which settings actually increase the system performance, we know nothing about the upper limit of ranking performance (given the results from our term recognizer). In order to assess the distance to this upper bound, we take the results of our best system and build a perfect ranking on top of it by pushing all true positives in front of all its false positives. The Figures 1 and 2 plot this information for varying cut-off limits: the lower limit of performance is given by the baseline (t-e0-m0), the upper limit is derived from our best setting for the respective metrics.

### Evaluation of relation ranking: TAP-10

The evaluation metrics of TAP-*k *is of utmost significance for our application scenario of database curation due to the fact that curators are not willing to sort out a large number of false positive relation candidates. For both data sets we take *k *= 10, which means that after having seen 10 false positives no further results are taken into consideration.

The upper part of Table 4 shows the feature-specific performance increase of TAP-10 for the PharmGKB. The type preference coefficient e1 improves the baseline most, followed by the application of ME. Note that metadata (tmc) improves results only modestly for the PharmGKB, in fact, using metadata without applying ME optimization performs worse than the baseline. A cap of 9 (c9) never results in the best increase for TAP-10 on the PharmGKB nor does it in any other ranking evaluation. However, applying a cap of 6 seems to be the best strategy for the PharmGKB. As the baseline for the PharmGKB is already well performing, the overall relative improvement is limited to 53%.

As shown for the CTD in the lower part of Table 4, the order of features leading to the highest performance is similar to the one from the PharmGKB. However, the addition of metadata has a much stronger impact for the CTD. One reason for that may be the use of MeSH terminology in the CTD dictionaries. Having frequency counts in the gold probability features (i.e. having a setting other than c1) leads to a relatively small performance increase. The best settings for the PharmGKB and the CTD only differ in the cap (c6 vs. c3), which supports the conclusion that the techniques are generally applicable. In the case of the CTD, the rather low baseline performance is improved by more than 134%.

The plots in Figure 1 show that the best setting for the PharmGKB not only performs better in terms of absolute TAP scores than the best setting for the CTD. Additionally, the best setting from the PharmGKB reduces the distance to the upper limit far more than the best setting for the CTD. One possible explanation for this fact may be given by the different distribution of articles containing a single relation: in the PharmGKB almost 40% of all articles contribute just one relation whereas in the CTD only about 22% do this.

### Evaluation of relation ranking: AUCiP/R

According to our application scenario we apply a cut-off limit of 50 relations to all evaluations of AUCiP/R. The upper part of Table 5 shows the feature-specific performance increase for the PharmGKB. In contrast to the TAP measure, the addition of metadata is more important, thus expressing the fact that AUCiP/R is more sensitive to the improvement of recall than TAP-*k*. Again, determining the best settings for the CTD is more straightforward than for the PharmGKB. Although the improvement for the different performance increase steps are statistically significant, there are only small differences between the top settings. Note that the top setting for TAP-10 and for AUCiP/R are different for the PharmGKB. In contrast, the lower part of Table 5 shows almost the same feature ranking for the CTD for both evaluation metrics. We regard the switch between the order of n3 and r1 not as important, given the fact that the lower improvement confidence interval CI*_l _*for n3 is much lower than the "random" empirical improvement of 0.0018. For the PharmGKB we achieve an overall improvement of 40%. For the CTD, which again has a much lower baseline, the best setting improves by 116%.

The plots in Figure 2 illustrate the dependency of AUCiP/R on recall. Note that whereas in Figure 1 the best settings for the PharmGKB seems apparently closer to the perfect ranking than the CTD, this difference is less prominent in terms of AUCiP/R.

### Evaluation of metadata contribution

The inclusion of metadata such as MeSH or chemical substances into the text mining procedure improves the overall performance of relation ranking. Although this information is widely available from PubMed (or directly from the publishers), it may be missing for some texts. In Tables 6 we show how the performance of the best settings decreases from missing metadata. For the PharmGKB the difference is modest (under 5%) if all metadata is discarded. For the CTD the TAP-10 score is almost 13% higher if metadata is used. This difference correlates with the coverage improvements for metadata inclusion as shown in Table 2.

**Table 6:** Evaluation of metadata contribution

PharmGKB data set TAP-10								
**setting**	**mean**	**sd**	**Δ**	**Δabs**	**Δrel**	**p value**	**ΔCI*_l_***	**Δrel*_bs_***

t-e1-r1-m1-n3-c6-s1	0.40	0.011						
tc-e1-r1-m1-n3-c6-s1	0.41	0.010	tc	+0.006	+1.4	9.8_-4_	+0.004	+1.4
tm-e1-r1-m1-n3-c6-s1	0.41	0.011	tm	+0.008	+2.0	2.0_-3_	+0.005	+3.4
tmc-e1-r1-m1-n3-c6-s1	0.42	0.009	tmc	+0.004	+1.1	2.0_-3_	+0.002	+4.5

**PharmGKB data set AUCiP/R**								

**setting**	**mean**	**sd**	**Δ**	**Δabs**	**Δrel**	**p value**	**ΔCI*_l_***	**Δrel*_bs_***

t-e1-r1-m1-n3-c0-s1	0.53	0.015						
tc-e1-r1-m1-n3-c0-s1	0.53	0.014	tc	+0.007	+1.4	2.0_-3_	+0.004	+1.4
tm-e1-r1-m1-n3-c0-s1	0.54	0.015	tm	+0.010	+1.8	2.0_-3_	+0.006	+3.2

**CTD data set TAP-10**								

**setting**	**mean**	**sd**	**Δ**	**Δabs**	**Δrel**	**p value**	**ΔCI*_l_***	**Δrel*_bs_***

t-r1-m1-n3-c3-s1	0.31	0.006						
tc-r1-m1-n3-c3-s1	0.33	0.005	tc	+0.022	+7.0	9.8_-4_	+0.019	+7.0
tm-r1-m1-n3-c3-s1	0.34	0.004	tm	+0.007	+2.0	9.8_-4_	+0.005	+9.2
tmc-r1-m1-n3-c3-s1	0.35	0.005	tmc	+0.011	+3.3	9.8_-4_	+0.010	+12.8

**CTD data set AUCiP/R**								

**setting**	**mean**	**sd**	**Δ**	**Δabs**	**Δrel**	**p value**	**ΔCI*_l_***	**Δrel*_bs_***

t-r1-m1-n3-c3-s1	0.40	0.008						
tc-r1-m1-n3-c3-s1	0.43	0.007	tc	+0.030	+7.5	9.8_-4_	+0.027	+7.5
tm-r1-m1-n3-c3-s1	0.45	0.006	tm	+0.014	+3.3	9.8_-4_	+0.012	+11.1
tmc-r1-m1-n3-c3-s1	0.46	0.007	tmc	+0.014	+3.0	9.8_-4_	+0.011	+14.5

This table shows the increase of performance for each step of inclusion of metadata as applied to best settings of TAP-10 and AUCiP/R. See Table 4 for a detailed explanation on the interpretation of the columns.

### Evaluation of Precision, Recall, and F-Measure

**Figure 3:**
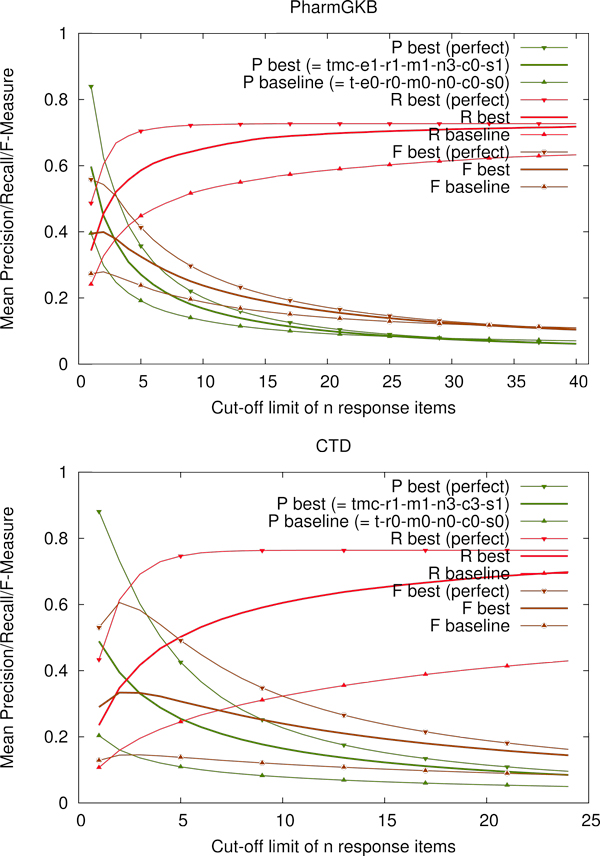
**Evaluation of Precision, Recall and F-Measure**. Mean macro-averaged results from the BioCreative evaluation tool. The horizontal axis shows the cut-off value limiting the number of hits that are evaluated by the tool. The vertical axis shows macro averaged results of precision (P), recall (R) and F-Measure (F) for our different approaches. Note that these results were computed by ignoring documents without hits in the system responses (this is the default setting for the BioCreative evaluations).

The plots in Figure 3 show the corresponding numbers as computed by the Biocreative evaluation tool for the best system settings as resulting from the TAP-10 evaluation. Note that for small cut-offs, precision is high, e.g. the first solution is a correct relation in almost 60% of all cases on average in the PharmGKB, and almost 50% in the CTD. However, precision drops quickly given the fact that there are not that many articles with more than 5 relations. For the PharmGKB the baseline performs better than the best system using cut-off limits *n *> 30, which could be an adverse effect that our training material is limited to articles with at most 24 relations.

### Evaluation of the estimation of gold probabilities

A substantial part of the performance of the maximum-entropy-based ranking depends on the proper estimation of the probability of an entity to be part of a true positive relation. Therefore, we evaluated the probability scores separately with regard to the experimental settings. Table 7 shows significant performance improvements by smoothing the feature counts (s1), by using metadata (tmc), and by applying the strongest normalization (n3). Applying a cap of 9 (c9) improves minimally but is not statistically significant. Note that the best setting for the gold probability does not carry over as the best setting for TAP oder AUCiP/R.

**Table 7:** Evaluation of performance increase of gold probability

PharmGKB data set								
**setting**	**mean**	**sd**	**Δ**	**Δabs**	**Δrel**	**p value**	**ΔCI*_l_***	**Δrel*_bs_***

t-m1-n0-c0-s0	0.85	0.003						
t-m1-n0-c0-s1	0.89	0.002	s1	+0.031	+3.7	9.8_-4_	+0.030	+3.7
tmc-m1-n0-c0-s1	0.90	0.002	tmc	+0.016	+1.8	9.8_-4_	+0.015	+5.5
tmc-m1-n3-c0-s1	0.91	0.002	n3	+0.009	+1.0	9.8_-4_	+0.008	+6.5
tmc-m1-n3-c9-s1	0.91	0.002	c9	+0.000	+0.0	3.5_-1_	+0.000	+6.5

**CTD data set**								

**setting**	**mean**	**sd**	**Δ**	**Δabs**	**Δrel**	**p value**	**ΔCI*_l_***	**Δrel*_bs_***

t-m1-n0-c0-s0	0.91	0.001						
t-m1-n0-c0-s1	0.94	0.000	s1	+0.028	+3.0	9.8_-4_	+0.027	+3.0
t-m1-n3-c0-s1	0.95	0.001	n3	+0.010	+1.1	9.8_-4_	+0.010	+4.2
tm-m1-n3-c0-s1	0.95	0.001	tm	+0.005	+0.5	9.8_-4_	+0.005	+4.7
tm-m1-n3-c6-s1	0.95	0.001	c6	+0.000	+0.0	1.2_-1_	+0.000	+4.7
tmc-m1-n3-c6-s1	0.95	0.001	tmc	+0.000	+0.0	5.0_-1_	+0.000	+4.7
tmc-m1-n3-c9-s1	0.95	0.001	c9	+0.000	+0.0	3.8_-1_	+0.000	+4.7

This table gives the corresponding overview of feature-wise performance increase as Table 4. See Table 4 for a detailed explanation on the interpretation of the columns. The performance of the gold probability shows the quality of the Maximum Entropy approach for the estimation of an entity being part of a relation from the gold standard.

### Usage in a curation environment

Advanced text mining techniques are now reaching a maturity level that makes them increasingly relevant for the process of curation of biomedical literature. As part of our research in this area we developed a curation system called "OntoGene Document INspector" (ODIN [21]) which interfaces with our OntoGene text mining pipeline (OG-RM). We have used a version of ODIN for our participation to the 'interactive curation' task (IAT) of the BioCreative III competition [22]. This was an informal task without a quantitative evaluation of the participating systems. However, the curators who used the system commented positively on its usability for a practical curation task.

**Figure 4:**
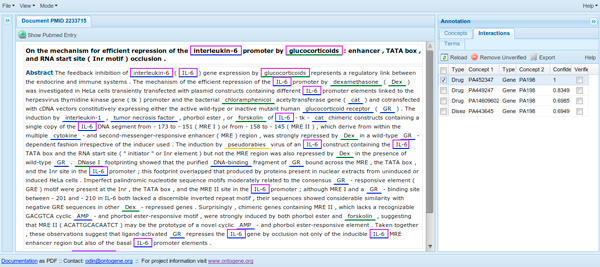
**ODIN curation interface**. Example of interaction with the ODIN system. Terms identified by the system are underlined in the abstract. Candidate relations are shown in the left-hand-side panel. Selecting a relation automatically highlights the terms in the document which correspond to the entities in the relation.

More recently, we have created a version of ODIN which allows inspection of abstracts automatically annotated with PharmGKB entities (the annotation is performed using OG-RM). Users can access either preprocessed documents, or enter any PubMed identifier and the corresponding abstract will be processed "on the fly". For the documents already contained in the PharmGKB it is also possible to compare the results of the system against the gold standard. The curator can inspect all entities annotated by the system, and easily modify them if needed (removing false positives with a simple click, or adding missed terms if necessary). The modified documents can be sent back for processing if desired, obtaining therefore modified candidate interactions. The user can also inspect the set of candidate interactions generated by the system, and act upon them just as on entities, i.e., confirm those which are correct, remove those which are incorrect. Candidate interactions are presented sorted according to the score which has been assigned to them by the text mining system, therefore the curator can choose to work with a small set of highly ranked candidates only, ignoring all the rest (see Figure 4). Recent user experiments using our curation environment, which makes use of the ranking proposed by the method described above, have shown positive results [23]. Additionally, a relation reranking on a CTD dataset, based on the approach described in this paper, has contributed to competitive results in the recent triage task (task 1) of the BioCreative 2012 shared task [24].

### Outlook

As a continuation of this work, we would like to estimate the number of relations to be found in a paper on the basis of its textual content. Being able to provide this information before or at the initial stages of the curation process would help the curators to decide at which point of the curation process it is most sensible to stop after having found a given number of correct relations. This is particularly relevant because documents differ greatly in the number of relations they describe, ranging from a single relation to several hundred ones in a few documents describing high-throughput experiments. In the PharmGKB we have observed that 40% of the documents contain only one relation, however they contribute less than 10% of all relations. Approx. 90% of the documents contain 10 or less relations, however these documents contain around 50% of all relations. So the remaining 10% of documents (which contributes more than 50% of the relations) have a much higher number of relations per document. In the CTD 23% of the documents contain only one relation and contribute to 2.2% of all relations. Approx. 90% of the documents contain 12 or less relations.

A possible limitation of the proposed approach is that it favors conservative assumptions, i.e. it privileges entities and relationships which have already been seen over totally new entities and relationships. The inclusion of contextual and linguistic features might help compensate for this bias. A further question left for future work concerns the use and impact of alternative term recognizers (e.g. BANNER [25], MetaMap [26]) and additional terminological resources [9,10].

## Conclusions

We have presented a simple and practical approach for the mining and ranking of pharmacogenomic and toxicogenomic relations, and evaluated this approach systematically against two different knowledge bases, the PharmGKB and the CTD. We have implemented a Maximum Entropy technique for the optimized ranking of candidate relations using a purely frequency-based text mining approach. In order to estimate the relevance of a relation candidate for a new article, we combine textual evidence from the article with the evidence derived from the large set of relations found in curated articles. Our experiments show that this approach is feasible, and our results might offer a useful baseline for further developments that apply more sophisticated techniques from the field of protein-protein interaction detection [27]. Whereas for the experiments described in this paper we use only simple frequency-based features, the next step is to include contextual [28,29] and linguistic [30] features. The Maximum Entropy technique we applied so far is ideally suited for doing this.

We have used existing tools to score the results and to provide reliable evaluation metrics, including not only the traditional Precision, Recall and F-Measure, but also the increasingly important measures of ranking quality, such as AUC iP/R or TAP-k. The evaluation shows that the reranking techniques described in this article bring a considerable improvement to the results.

Finally, we have briefly mentioned the usage of these results within an assisted curation environment (ODIN), which is discussed more extensively in separate publications [23,24]. The experience from these experiments suggests that the usability of a curation environment is enhanced considerably by the presentation of properly ranked relation candidates.

## Competing interests

The authors declare that they have no competing interests.

## Authors' contributions

Fabio Rinaldi conceived the general experimental setting described in this article. Simon Clematide devised and implemented the Maximum Entropy approach for reranking of candidate interactions, and conducted all the experiments and evaluations described in this paper. This research takes place within the scope of the SASEBio project (Semi-Automated Semantic Enrichment of the Biomedical Literature, SNF 100014-118396/1) awarded to Fabio Rinaldi.
